# Bis(1,10-phenanthroline-5,6-dione-κ^2^ *N*,*N*′)silver(I) 2-hy­droxy-3,5-dinitro­benzoate

**DOI:** 10.1107/S1600536811053785

**Published:** 2011-12-21

**Authors:** Shen-Tang Wang, Guang-Bo Che, Chun-Bo Liu, Xing Wang, Ling Liu

**Affiliations:** a School of Chemistry and Chemical Engineering, Jiangsu University, Zhenjiang 212013, People’s Republic of China

## Abstract

In the cation of the title salt, [Ag(C_12_H_6_N_2_O_2_)_2_](C_7_H_3_N_2_O_7_), the Ag^I^ atom is coordinated in a distorted tetra­hedral geometry by four N atoms from two 1,10-phenanthroline-5,6-dione ligands, while the 3,5-dinitro­salicylate anion has only a short contact [2.847 (6) Å] between one of its O atoms and the Ag^I^ atom. The dihedral angle between the two 1,10-phenanthroline-5,6-dione ligands is 58.4 (1)°. There is an intra­molecular O—H⋯O hydrogen bond in the 3,5-dinitro­salicylate anion.

## Related literature

For general background to the structures and potential applications of supra­molecular architectures with 1,10-phenantroline-5,6-dione and 3,5-dinitro­salicylic acid, see: Hiort *et al.* (1993 ▶); Song *et al.* (2007 ▶); Che *et al.* (2008 ▶); Onuegbu *et al.* (2009 ▶). For the synthesis of the 1,10-phenantroline-5,6-dione ligand, see: Dickeson & Sumers (1970 ▶).
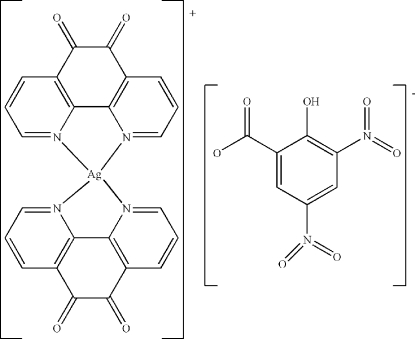


## Experimental

### 

#### Crystal data

[Ag(C_12_H_6_N_2_O_2_)_2_](C_7_H_3_N_2_O_7_)
*M* *_r_* = 755.36
Monoclinic, 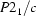

*a* = 11.757 (2) Å
*b* = 18.297 (4) Å
*c* = 13.223 (3) Å
β = 103.91 (3)°
*V* = 2761.1 (11) Å^3^
*Z* = 4
Mo *K*α radiation
μ = 0.81 mm^−1^
*T* = 174 K
0.30 × 0.24 × 0.20 mm

#### Data collection

Bruker SMART diffractometer
Absorption correction: multi-scan (*SADABS*; Bruker, 2002 ▶) *T*_min_ = 0.780, *T*_max_ = 0.910
12726 measured reflections
5059 independent reflections
3914 reflections with *I* > 2σ(*I*)
*R*_int_ = 0.052

#### Refinement

*R*[*F* ^2^ > 2σ(*F* ^2^)] = 0.083
*wR*(*F* ^2^) = 0.163
*S* = 1.11
5013 reflections
442 parameters
22 restraints
H-atom parameters constrained
Δρ_max_ = 1.11 e Å^−3^
Δρ_min_ = −0.72 e Å^−3^

### 

Data collection: *SMART* (Bruker, 2007 ▶); cell refinement: *SAINT* (Bruker, 2007 ▶); data reduction: *SAINT*; program(s) used to solve structure: *SHELXS97* (Sheldrick, 2008 ▶); program(s) used to refine structure: *SHELXL97* (Sheldrick, 2008 ▶); molecular graphics: *SHELXTL* (Sheldrick, 2008 ▶) and *DIAMOND* (Brandenburg, 1999 ▶); software used to prepare material for publication: *SHELXTL*.

## Supplementary Material

Crystal structure: contains datablock(s) global, I. DOI: 10.1107/S1600536811053785/vn2025sup1.cif

Structure factors: contains datablock(s) I. DOI: 10.1107/S1600536811053785/vn2025Isup2.hkl

Additional supplementary materials:  crystallographic information; 3D view; checkCIF report

## Figures and Tables

**Table 1:** Selected bond lengths (Å)

Ag1—N1	2.400 (6)
Ag1—N2	2.351 (6)
Ag1—N3	2.337 (6)
Ag1—N4	2.377 (6)

**Table 2:** Hydrogen-bond geometry (Å, °)

*D*—H⋯*A*	*D*—H	H⋯*A*	*D*⋯*A*	*D*—H⋯*A*
O7—H7⋯O8	0.82	1.71	2.457 (9)	151

**Fig. 1.**
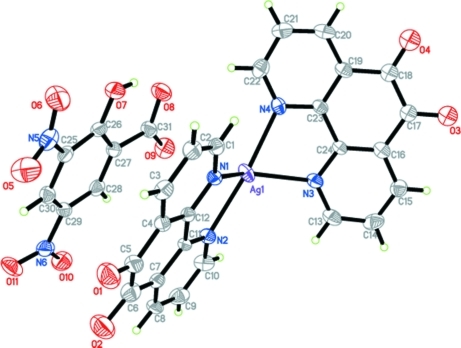
The molecular structure of the title compound with the atom numbering scheme. Displacement ellipsoids are drawn at the 30% probability level. All H atoms are presented as a small spheres of arbitrary radius.

**Fig. 2.**
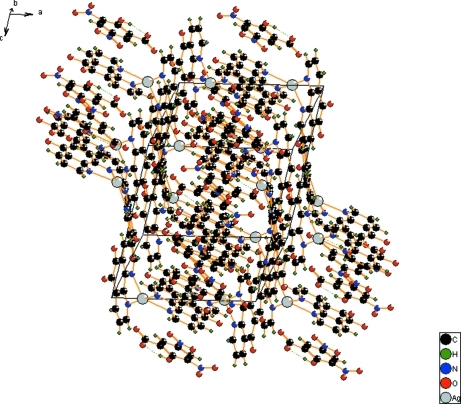
A view of the crystal packing of the title compound, showing the O–H···O hydrogen bonds interaction.

## supplementary crystallographic information

### Comment

The design and construction of supramolecular architectures have received considerable attention in recent years, mostly motivated by their intriguing structural features and potential applications in molecular adsorption, molecular sensing, magnetism, catalysis and non-linear optics (Che *et al.*, 2008). Metal complexes with 1,10-phenantroline-5,6-dione (L) and 3,5-dinitrosalicylic acid as important ligands for the construction of metal-organic supramolecular architectures have been increasingly studied over recent years (Hiort *et al.*, 1993; Onuegbu *et al.*, 2009; Song *et al.*, 2007). We report herein on the crystal structure of the title compound (Fig. 1). The molecular structure of the title compound, is made up of one [AgL_2_]^+^ cation and one 3,5-dinitrosalicylate anion. The Ag^I^ atom is surrounded by four N atoms from two 1,10-phenanthroline-5,6-dione ligands, while the 3,5-dinitrosalicylate ligand is uncoordinated. In the compound the dihedral angle between the phendione ligand A (C1-C12, N1, N2, O1 and O2) and B (C13-C24, N3, N4, O3, and O4) is 58.4 (1)°. The dihedral angle between B and 3,5-dinitrosalicylate ligand C (C25-C31, N5, N6, O5-O11) is 56.1 (2)°. The dihedral angle between A and C is 2.4 (9)°, suggesting that the planes of rings A and C are almost parallel. In addition, in the 3,5-dinitrosalicylate ligand, there is one intramolecular O–H···O hydrogen bond (Fig. 2).

### Experimental

The L ligand was synthesized according to the literature method (Dickeson & Sumers, 1970). The title compound was synthesized under hydrothermal conditions. A mixture of L (0.042 g, 0.2 mmol), 3,5-dinitrosalicylic acid (0.046 g, 0.2 mmol), AgNO_3_ (0.034 g, 0.2 mmol) and water (10 mL) was placed in a 25 mL Teflon-lined autoclave and heated for 3 days at 433 K under autogenous pressure. Upon cooling and opening the bomb, yellow block-shaped crystals were obtained, then washed with water and dried in air.

### Refinement

All H atoms on C atoms were positioned geometrically and refined as riding atoms, with (C—H = 0.93 Å) and refined as riding, with *U*_iso_(H) = 1.2 *U*_eq_(C). The hydrogen atom of the hydroxyl group was located in a difference Fourier map, and was refined with a suitable O—H distance restraint; *U*_iso_(H) = 1.5 *U*_eq_(O). The geometry of the aromatic rings was regularized using distance and planariety restraints.

### Crystal data

**Table:**

C_24_H_12_AgN_4_O_4_·C_7_H_3_N_2_O_7_	*F*(000) = 1512
*M**_r_* = 755.36	*D*_x_ = 1.817 Mg m^−^^3^
Monoclinic, *P*2_1_/*c*	Mo *K*α radiation, λ = 0.71073 Å
Hall symbol: -P 2ybc	Cell parameters from 5197 reflections
*a* = 11.757 (2) Å	θ = 3.2–25.4°
*b* = 18.297 (4) Å	µ = 0.81 mm^−^^1^
*c* = 13.223 (3) Å	*T* = 174 K
β = 103.91 (3)°	Prism, yellow
*V* = 2761.1 (11) Å^3^	0.3 × 0.24 × 0.2 mm
*Z* = 4	

### Data collection

**Table:**

Oxford Diffraction Gemini R Ultra diffractometer	5059 independent reflections
Radiation source: fine-focus sealed tube	3914 reflections with *I* > 2σ(*I*)
graphite	*R*_int_ = 0.052
ω scans	θ_max_ = 25.4°, θ_min_ = 3.2°
Absorption correction: multi-scan *SADABS* (Bruker, 2002)	*h* = −14→11
*T*_min_ = 0.780, *T*_max_ = 0.910	*k* = −17→22
12726 measured reflections	*l* = −15→13

### Refinement

**Table:**

Refinement on *F*^2^	Primary atom site location: structure-invariant direct methods
Least-squares matrix: full	Secondary atom site location: difference Fourier map
*R*[*F*^2^ > 2σ(*F*^2^)] = 0.083	Hydrogen site location: inferred from neighbouring sites
*wR*(*F*^2^) = 0.163	H-atom parameters constrained
*S* = 1.11	*w* = 1/[σ^2^(*F*_o_^2^) + (0.0354*P*)^2^ + 13.4705*P*] where *P* = (*F*_o_^2^ + 2*F*_c_^2^)/3
5013 reflections	(Δ/σ)_max_ < 0.001
442 parameters	Δρ_max_ = 1.11 e Å^−^^3^
22 restraints	Δρ_min_ = −0.72 e Å^−^^3^

### Special details

**Table:**

Geometry. All esds (except the esd in the dihedral angle between two l.s. planes) are estimated using the full covariance matrix. The cell esds are taken into account individually in the estimation of esds in distances, angles and torsion angles; correlations between esds in cell parameters are only used when they are defined by crystal symmetry. An approximate (isotropic) treatment of cell esds is used for estimating esds involving l.s. planes.
Refinement. Refinement of F^2^ against ALL reflections. The weighted R-factor wR and goodness of fit S are based on F^2^, conventional R-factors R are based on F, with F set to zero for negative F^2^. The threshold expression of F^2^ > 2sigma(F^2^) is used only for calculating R-factors(gt) etc. and is not relevant to the choice of reflections for refinement. R-factors based on F^2^ are statistically about twice as large as those based on F, and R- factors based on ALL data will be even larger.

### Fractional atomic coordinates and isotropic or equivalent isotropic displacement parameters (Å^2^)

**Table:**

	*x*	*y*	*z*	*U*_iso_*/*U*_eq_	
Ag1	0.21081 (6)	0.44038 (3)	0.52487 (5)	0.0540 (2)	
C1	0.4793 (7)	0.5137 (4)	0.6222 (5)	0.0435 (18)	
H1	0.4399	0.5580	0.6074	0.052*	
C2	0.5976 (7)	0.5157 (4)	0.6690 (6)	0.048 (2)	
H2	0.6371	0.5599	0.6845	0.058*	
C3	0.6552 (7)	0.4497 (4)	0.6920 (6)	0.0470 (19)	
H3	0.7345	0.4488	0.7248	0.056*	
C4	0.5941 (6)	0.3844 (4)	0.6659 (5)	0.0380 (16)	
C5	0.6515 (9)	0.3155 (4)	0.6883 (7)	0.062 (2)	
C6	0.5895 (7)	0.2505 (5)	0.6710 (7)	0.063 (2)	
C7	0.4678 (6)	0.2544 (4)	0.6244 (5)	0.0431 (18)	
C8	0.3994 (8)	0.1885 (4)	0.6037 (6)	0.053 (2)	
H8	0.4340	0.1433	0.6229	0.064*	
C9	0.2836 (9)	0.1930 (5)	0.5557 (7)	0.063 (2)	
H9	0.2388	0.1508	0.5403	0.075*	
C10	0.2342 (7)	0.2594 (5)	0.5305 (7)	0.055 (2)	
H10	0.1552	0.2613	0.4966	0.066*	
C11	0.4090 (6)	0.3201 (3)	0.5978 (5)	0.0338 (16)	
C12	0.4738 (5)	0.3869 (3)	0.6196 (5)	0.0335 (16)	
C13	0.0848 (7)	0.4147 (4)	0.2775 (7)	0.055 (2)	
H13	0.1080	0.3666	0.2933	0.066*	
C14	0.0374 (8)	0.4324 (5)	0.1743 (7)	0.061 (2)	
H14	0.0287	0.3972	0.1223	0.073*	
C15	0.0034 (7)	0.5036 (5)	0.1506 (6)	0.053 (2)	
H15	−0.0298	0.5170	0.0820	0.064*	
C16	0.0190 (6)	0.5564 (4)	0.2312 (5)	0.0432 (18)	
C17	−0.0122 (5)	0.6312 (4)	0.2098 (5)	0.052 (2)	
C18	−0.0077 (7)	0.6805 (4)	0.2948 (6)	0.055 (2)	
C19	0.0460 (6)	0.6571 (4)	0.3993 (6)	0.0447 (18)	
C20	0.0688 (7)	0.7058 (4)	0.4853 (7)	0.055 (2)	
H20	0.0483	0.7548	0.4749	0.067*	
C21	0.1205 (8)	0.6816 (5)	0.5829 (7)	0.059 (2)	
H21	0.1348	0.7131	0.6398	0.071*	
C22	0.1506 (7)	0.6090 (5)	0.5945 (6)	0.056 (2)	
H22	0.1865	0.5925	0.6611	0.067*	
C23	0.0809 (6)	0.5844 (3)	0.4175 (5)	0.0369 (16)	
C24	0.0632 (5)	0.5330 (3)	0.3334 (5)	0.0322 (15)	
C25	0.6149 (7)	0.4146 (4)	0.9278 (5)	0.0419 (18)	
C26	0.5083 (7)	0.4530 (4)	0.8867 (5)	0.0411 (18)	
C27	0.4075 (6)	0.4103 (4)	0.8430 (5)	0.0388 (17)	
C28	0.4092 (7)	0.3351 (4)	0.8460 (5)	0.0433 (18)	
H28	0.3421	0.3080	0.8181	0.052*	
C29	0.5165 (8)	0.3002 (4)	0.8928 (5)	0.0434 (19)	
C30	0.6177 (7)	0.3387 (4)	0.9327 (6)	0.0435 (18)	
H30	0.6871	0.3143	0.9625	0.052*	
C31	0.2956 (8)	0.4467 (5)	0.7881 (6)	0.050 (2)	
N1	0.4178 (5)	0.4524 (3)	0.5970 (4)	0.0347 (13)	
N2	0.2931 (5)	0.3228 (3)	0.5518 (5)	0.0399 (14)	
N3	0.0992 (5)	0.4629 (3)	0.3562 (5)	0.0422 (15)	
N4	0.1319 (5)	0.5605 (3)	0.5164 (5)	0.0423 (14)	
N5	0.7268 (7)	0.4516 (5)	0.9629 (5)	0.0587 (19)	
N6	0.5177 (8)	0.2213 (4)	0.9005 (5)	0.0560 (19)	
O1	0.7645 (7)	0.3121 (4)	0.7270 (6)	0.088 (2)	
O2	0.6419 (7)	0.1890 (4)	0.6988 (6)	0.096 (2)	
O3	−0.0430 (5)	0.6542 (3)	0.1162 (4)	0.0700 (18)	
O4	−0.0502 (6)	0.7445 (3)	0.2780 (5)	0.080 (2)	
O5	0.8165 (6)	0.4147 (4)	0.9745 (6)	0.091 (2)	
O6	0.7295 (6)	0.5175 (4)	0.9763 (6)	0.085 (2)	
O7	0.5032 (6)	0.5236 (3)	0.8852 (4)	0.0608 (16)	
H7	0.4365	0.5366	0.8565	0.091*	
O8	0.2947 (6)	0.5172 (3)	0.7949 (5)	0.0684 (17)	
O9	0.2133 (5)	0.4109 (4)	0.7368 (5)	0.0641 (16)	
O10	0.4296 (7)	0.1876 (3)	0.8584 (5)	0.0713 (19)	
O11	0.6097 (6)	0.1916 (3)	0.9511 (5)	0.0684 (18)	

### Atomic displacement parameters (Å^2^)

**Table:**

	*U*^11^	*U*^22^	*U*^33^	*U*^12^	*U*^13^	*U*^23^
Ag1	0.0472 (4)	0.0491 (4)	0.0609 (4)	0.0150 (3)	0.0036 (3)	0.0149 (3)
C1	0.056 (5)	0.038 (4)	0.039 (4)	−0.007 (4)	0.015 (4)	0.001 (3)
C2	0.054 (5)	0.046 (5)	0.048 (5)	−0.011 (4)	0.019 (4)	−0.001 (4)
C3	0.033 (4)	0.065 (6)	0.042 (4)	−0.003 (4)	0.008 (3)	−0.006 (4)
C4	0.036 (4)	0.043 (4)	0.037 (4)	0.007 (3)	0.013 (3)	0.001 (3)
C5	0.084 (7)	0.059 (5)	0.049 (5)	0.022 (5)	0.028 (5)	0.008 (4)
C6	0.067 (5)	0.068 (6)	0.057 (5)	0.007 (5)	0.019 (4)	−0.001 (5)
C7	0.056 (4)	0.045 (4)	0.033 (4)	0.005 (4)	0.020 (3)	0.001 (3)
C8	0.085 (5)	0.031 (4)	0.049 (5)	0.014 (4)	0.026 (4)	0.008 (4)
C9	0.077 (5)	0.053 (5)	0.064 (6)	−0.012 (5)	0.027 (4)	−0.010 (5)
C10	0.043 (5)	0.050 (5)	0.072 (6)	−0.002 (4)	0.014 (4)	−0.009 (4)
C11	0.044 (4)	0.027 (4)	0.031 (4)	0.008 (3)	0.011 (3)	0.001 (3)
C12	0.041 (4)	0.031 (4)	0.030 (4)	−0.001 (3)	0.011 (3)	0.002 (3)
C13	0.051 (5)	0.036 (4)	0.072 (6)	−0.001 (4)	0.005 (4)	−0.002 (4)
C14	0.056 (6)	0.057 (6)	0.063 (6)	−0.002 (4)	0.002 (4)	−0.024 (5)
C15	0.047 (5)	0.060 (6)	0.047 (5)	−0.001 (4)	0.003 (4)	−0.002 (4)
C16	0.032 (4)	0.042 (4)	0.053 (5)	0.000 (3)	0.006 (3)	0.004 (4)
C17	0.041 (5)	0.053 (5)	0.058 (5)	0.003 (4)	0.002 (4)	0.019 (4)
C18	0.041 (5)	0.049 (5)	0.071 (6)	−0.004 (4)	0.004 (4)	0.006 (5)
C19	0.038 (4)	0.037 (4)	0.058 (5)	0.001 (3)	0.008 (4)	0.001 (4)
C20	0.045 (5)	0.035 (4)	0.084 (7)	0.001 (4)	0.012 (5)	−0.008 (4)
C21	0.052 (5)	0.055 (5)	0.069 (6)	0.003 (4)	0.012 (5)	−0.017 (5)
C22	0.047 (5)	0.070 (6)	0.047 (5)	−0.002 (4)	0.004 (4)	−0.005 (4)
C23	0.028 (4)	0.036 (4)	0.045 (4)	0.003 (3)	0.005 (3)	0.002 (3)
C24	0.022 (3)	0.029 (4)	0.045 (4)	0.000 (3)	0.006 (3)	0.004 (3)
C25	0.056 (5)	0.040 (4)	0.029 (4)	−0.010 (4)	0.008 (3)	−0.003 (3)
C26	0.061 (5)	0.033 (4)	0.033 (4)	0.001 (4)	0.018 (4)	−0.003 (3)
C27	0.045 (5)	0.039 (4)	0.035 (4)	0.001 (3)	0.016 (3)	0.003 (3)
C28	0.061 (5)	0.042 (4)	0.032 (4)	−0.008 (4)	0.019 (4)	−0.002 (3)
C29	0.071 (6)	0.030 (4)	0.035 (4)	0.006 (4)	0.024 (4)	0.006 (3)
C30	0.047 (5)	0.046 (5)	0.038 (4)	0.004 (4)	0.013 (3)	0.004 (3)
C31	0.060 (6)	0.049 (5)	0.048 (5)	0.014 (4)	0.024 (4)	0.011 (4)
N1	0.036 (3)	0.032 (3)	0.035 (3)	0.002 (3)	0.008 (3)	0.000 (3)
N2	0.038 (4)	0.037 (3)	0.045 (3)	0.000 (3)	0.009 (3)	−0.001 (3)
N3	0.038 (4)	0.032 (3)	0.053 (4)	0.000 (3)	0.004 (3)	−0.002 (3)
N4	0.037 (3)	0.043 (4)	0.046 (4)	0.003 (3)	0.007 (3)	0.006 (3)
N5	0.059 (5)	0.071 (6)	0.042 (4)	−0.011 (4)	0.006 (3)	−0.007 (4)
N6	0.091 (6)	0.039 (4)	0.046 (4)	0.003 (4)	0.032 (4)	0.003 (3)
O1	0.081 (5)	0.099 (6)	0.084 (5)	0.033 (4)	0.020 (4)	0.021 (4)
O2	0.086 (6)	0.079 (5)	0.110 (6)	0.022 (4)	−0.003 (4)	0.000 (4)
O3	0.067 (4)	0.071 (4)	0.067 (4)	0.003 (3)	0.005 (3)	0.024 (3)
O4	0.097 (5)	0.040 (4)	0.094 (5)	0.018 (3)	0.005 (4)	0.016 (3)
O5	0.055 (5)	0.099 (6)	0.113 (6)	−0.004 (4)	0.008 (4)	−0.033 (5)
O6	0.089 (5)	0.050 (4)	0.098 (5)	−0.024 (4)	−0.016 (4)	0.002 (4)
O7	0.086 (5)	0.039 (3)	0.058 (4)	0.000 (3)	0.019 (3)	−0.001 (3)
O8	0.080 (5)	0.057 (4)	0.073 (4)	0.022 (3)	0.028 (3)	0.010 (3)
O9	0.041 (4)	0.081 (4)	0.069 (4)	0.004 (3)	0.012 (3)	−0.002 (3)
O10	0.110 (6)	0.039 (3)	0.066 (4)	−0.015 (4)	0.025 (4)	−0.003 (3)
O11	0.088 (5)	0.042 (3)	0.083 (4)	0.017 (3)	0.036 (4)	0.021 (3)

### Geometric parameters (Å, °)

**Table:**

Ag1—N1	2.400 (6)	C16—C24	1.394 (7)
Ag1—N2	2.351 (6)	C16—C17	1.428 (7)
Ag1—N3	2.337 (6)	C17—O3	1.275 (7)
Ag1—N4	2.377 (6)	C17—C18	1.432 (8)
Ag1—O9	2.847 (6)	C18—O4	1.272 (9)
C1—N1	1.333 (9)	C18—C19	1.438 (8)
C1—C2	1.380 (11)	C19—C23	1.397 (7)
C1—H1	0.9300	C19—C20	1.419 (11)
C2—C3	1.383 (11)	C20—C21	1.361 (12)
C2—H2	0.9300	C20—H20	0.9300
C3—C4	1.394 (10)	C21—C22	1.374 (12)
C3—H3	0.9300	C21—H21	0.9300
C4—C12	1.400 (7)	C22—N4	1.340 (10)
C4—C5	1.426 (8)	C22—H22	0.9300
C5—O1	1.304 (11)	C23—N4	1.373 (8)
C5—C6	1.385 (8)	C23—C24	1.433 (7)
C6—O2	1.293 (10)	C24—N3	1.362 (8)
C6—C7	1.417 (8)	C25—C30	1.390 (10)
C7—C11	1.390 (7)	C25—C26	1.426 (10)
C7—C8	1.438 (11)	C25—N5	1.454 (10)
C8—C9	1.358 (12)	C26—O7	1.292 (8)
C8—H8	0.9300	C26—C27	1.421 (10)
C9—C10	1.353 (12)	C27—C28	1.377 (10)
C9—H9	0.9300	C27—C31	1.498 (11)
C10—N2	1.347 (10)	C28—C29	1.416 (11)
C10—H10	0.9300	C28—H28	0.9300
C11—N2	1.353 (8)	C29—C30	1.374 (11)
C11—C12	1.432 (7)	C29—N6	1.448 (10)
C12—N1	1.365 (8)	C30—H30	0.9300
C13—N3	1.343 (10)	C31—O9	1.228 (10)
C13—C14	1.383 (12)	C31—O8	1.293 (10)
C13—H13	0.9300	N5—O6	1.218 (9)
C14—C15	1.376 (11)	N5—O5	1.230 (9)
C14—H14	0.9300	N6—O10	1.218 (9)
C15—C16	1.417 (10)	N6—O11	1.252 (9)
C15—H15	0.9300	O7—H7	0.8200
			
N3—Ag1—N2	115.0 (2)	O4—C18—C19	120.4 (7)
N3—Ag1—N4	70.7 (2)	C17—C18—C19	119.4 (7)
N2—Ag1—N4	174.1 (2)	C23—C19—C20	117.9 (7)
N3—Ag1—N1	130.3 (2)	C23—C19—C18	119.5 (7)
N2—Ag1—N1	71.5 (2)	C20—C19—C18	122.5 (7)
N4—Ag1—N1	106.3 (2)	C21—C20—C19	120.8 (7)
N3—Ag1—O9	147.53 (19)	C21—C20—H20	119.6
N2—Ag1—O9	76.74 (19)	C19—C20—H20	119.6
N4—Ag1—O9	97.64 (19)	C20—C21—C22	117.7 (8)
N1—Ag1—O9	81.75 (18)	C20—C21—H21	121.2
N1—C1—C2	124.2 (7)	C22—C21—H21	121.2
N1—C1—H1	117.9	N4—C22—C21	124.4 (8)
C2—C1—H1	117.9	N4—C22—H22	117.8
C1—C2—C3	117.6 (7)	C21—C22—H22	117.8
C1—C2—H2	121.2	N4—C23—C19	120.7 (6)
C3—C2—H2	121.2	N4—C23—C24	118.6 (6)
C2—C3—C4	119.9 (7)	C19—C23—C24	120.6 (6)
C2—C3—H3	120.0	N3—C24—C16	121.9 (6)
C4—C3—H3	120.0	N3—C24—C23	117.9 (6)
C3—C4—C12	119.1 (6)	C16—C24—C23	120.1 (6)
C3—C4—C5	121.0 (7)	C30—C25—C26	121.4 (7)
C12—C4—C5	119.9 (7)	C30—C25—N5	116.1 (7)
O1—C5—C6	118.0 (7)	C26—C25—N5	122.5 (7)
O1—C5—C4	120.7 (8)	O7—C26—C27	120.8 (7)
C6—C5—C4	121.3 (9)	O7—C26—C25	122.2 (7)
O2—C6—C5	120.3 (8)	C27—C26—C25	117.0 (6)
O2—C6—C7	122.0 (8)	C28—C27—C26	122.2 (7)
C5—C6—C7	117.8 (8)	C28—C27—C31	117.6 (7)
C11—C7—C6	122.8 (7)	C26—C27—C31	120.2 (7)
C11—C7—C8	117.1 (7)	C27—C28—C29	117.9 (7)
C6—C7—C8	120.1 (7)	C27—C28—H28	121.0
C9—C8—C7	119.4 (7)	C29—C28—H28	121.0
C9—C8—H8	120.3	C30—C29—C28	122.3 (7)
C7—C8—H8	120.3	C30—C29—N6	119.4 (8)
C10—C9—C8	119.5 (8)	C28—C29—N6	118.2 (8)
C10—C9—H9	120.3	C29—C30—C25	119.0 (7)
C8—C9—H9	120.3	C29—C30—H30	120.5
N2—C10—C9	123.6 (8)	C25—C30—H30	120.5
N2—C10—H10	118.2	O9—C31—O8	123.5 (8)
C9—C10—H10	118.2	O9—C31—C27	120.9 (8)
N2—C11—C7	122.1 (6)	O8—C31—C27	115.5 (8)
N2—C11—C12	119.3 (5)	C1—N1—C12	118.7 (6)
C7—C11—C12	118.6 (6)	C1—N1—Ag1	127.8 (5)
N1—C12—C4	120.6 (6)	C12—N1—Ag1	113.4 (4)
N1—C12—C11	119.9 (6)	C10—N2—C11	118.3 (6)
C4—C12—C11	119.5 (6)	C10—N2—Ag1	125.8 (5)
N3—C13—C14	123.8 (8)	C11—N2—Ag1	115.8 (4)
N3—C13—H13	118.1	C13—N3—C24	118.2 (6)
C14—C13—H13	118.1	C13—N3—Ag1	124.2 (5)
C15—C14—C13	118.2 (8)	C24—N3—Ag1	116.8 (4)
C15—C14—H14	120.9	C22—N4—C23	118.5 (6)
C13—C14—H14	120.9	C22—N4—Ag1	125.9 (5)
C14—C15—C16	119.8 (7)	C23—N4—Ag1	114.5 (4)
C14—C15—H15	120.1	O6—N5—O5	122.3 (8)
C16—C15—H15	120.1	O6—N5—C25	119.8 (8)
C24—C16—C15	118.0 (6)	O5—N5—C25	117.8 (8)
C24—C16—C17	120.3 (6)	O10—N6—O11	123.8 (7)
C15—C16—C17	121.7 (6)	O10—N6—C29	118.7 (8)
O3—C17—C18	120.3 (7)	O11—N6—C29	117.6 (8)
O3—C17—C16	120.4 (7)	C26—O7—H7	109.5
C18—C17—C16	119.3 (6)	C31—O9—Ag1	105.3 (5)
O4—C18—C17	120.2 (7)		

### Hydrogen-bond geometry (Å, °)

**Table:**

*D*—H···*A*	*D*—H	H···*A*	*D*···*A*	*D*—H···*A*
O7—H7···O8	0.82	1.71	2.457 (9)	151.
